# Determination of Autoantibody Isotypes Increases the Sensitivity of Serodiagnostics in Rheumatoid Arthritis

**DOI:** 10.3389/fimmu.2018.00876

**Published:** 2018-04-24

**Authors:** Daniela Sieghart, Alexander Platzer, Paul Studenic, Farideh Alasti, Maresa Grundhuber, Sascha Swiniarski, Thomas Horn, Helmuth Haslacher, Stephan Blüml, Josef Smolen, Günter Steiner

**Affiliations:** ^1^Division of Rheumatology, Department of Internal Medicine III, Medical University of Vienna, Vienna, Austria; ^2^Thermo Fisher Scientific, Phadia GmbH, Freiburg, Germany; ^3^Thermo Fisher Scientific, Phadia Austria GmbH, Vienna, Austria; ^4^Department of Laboratory Medicine, Medical University of Vienna, Vienna, Austria

**Keywords:** autoantibodies, rheumatoid factor, anti-citrullinated protein antibodies, RA33 antibodies, immunoglobulin isotypes, rheumatoid arthritis

## Abstract

Anti-citrullinated protein antibodies (ACPA) and rheumatoid factor (RF) are the most commonly used diagnostic markers of rheumatoid arthritis (RA). These antibodies are predominantly of the immunoglobulin (Ig) M (RF) or IgG (ACPA) isotype. Other subtypes of both antibodies—particularly IgA isotypes and other autoantibodies—such as RA33 antibodies—have been repeatedly reported but their diagnostic value has still not been fully elucidated. Here, we investigated the prevalence of IgA, IgG, and IgM subtypes of RF, ACPA, and RA33 antibodies in patients with RA. To determine the diagnostic specificity and sensitivity sera from 290 RA patients (165 early and 125 established disease), 261 disease controls and 100 healthy subjects were tested for the presence of IgA, IgG, and IgM isotypes of RF, ACPA, and RA33 by EliA™ platform (Phadia AB, Uppsala, Sweden). The most specific antibodies were IgG-ACPA, IgA-ACPA, and IgG-RF showing specificities >98%, closely followed by IgG- and IgA-RA33 while IgM subtypes were somewhat less specific, ranging from 95.8% (RA33) to 90% (RF). On the other hand, IgM-RF was the most sensitive subtype (65%) followed by IgG-ACPA (59.5%) and IgA-RF (50.7%). Other subtypes were less sensitive ranging from 35 (IgA-ACPA) to 6% (IgA-RA33). RA33 antibodies as well as IgA-RF and IgA-ACPA were found to increase the diagnostic sensitivity of serological testing since they were detected also in seronegative patients reducing their number from 109 to 85. Moreover, analyzing IgM-RF by EliA™ proved more sensitive than measuring RF by nephelometry and further reduced the number of seronegative patients to 76 individuals. Importantly, among antibody positive individuals, RA patients were found having significantly more antibodies (≥3) than disease controls which generally showed one or two antibody species. Thus, increasing the number of autoantibodies in serological routine testing provides valuable additional information allowing to better distinguish between RA and other rheumatic disorders, also in patients not showing antibodies in current routine diagnostics. In conclusion, testing for multiple autoantibody specificities increases the diagnostic power of autoimmune diagnostics and could further support physicians in clinical decision-making.

## Introduction

Rheumatoid arthritis (RA) is a systemic autoimmune disease characterized by chronic joint inflammation which leads to structural damage of bone and cartilage (1). Besides joint inflammation, duration of symptoms and acute-phase reactants, autoantibodies—rheumatoid factor (RF) and anti-citrullinated protein antibodies (ACPA)—are important diagnostic tools that are also used for classification of RA (2). RF is directed against the Fc portion of immunoglobulin (Ig) G and is usually measured by nephelometry, which captures all classes of Igs but mainly large molecules like IgM. ACPA are the most specific markers for RA and like RF appear early in the disease process and may precede clinical symptoms by several years (3). They are predominantly of the IgG isotype and are commonly measured by assays employing a cyclic citrullinated peptide (CCP) as antigen.

Despite the high sensitivity of the assays used in routine diagnostics still about one-third of RA patients are negative for IgG-ACPA and RF. It has been proposed by several authors that additional testing for other RF and ACPA isotypes—particularly IgA—might increase the sensitivity of RA serodiagnostics (4–8) or predict the development of disease (9), but clear-cut evidence is still scarce. Therefore, routine diagnostics are commonly restricted to measuring IgG-ACPA (usually by ELISA) and the determination of IgM-RF. RA33 antibodies (which are directed to the nuclear antigen hnRNP-A2/B1) were also found to be fairly specific for RA and testing for RA33 antibodies could be of additional diagnostic usefulness because they are also detected in RF/ACPA negative RA patients. However, published data on RA33 antibodies so far refer only to the IgG isotype and their use in routine diagnostics has not yet been widely established (10–13).

It was therefore the aim of this study to measure the IgG, IgA, and IgM isotypes of RF, ACPA, and RA33 in patients with RA and related rheumatic diseases in order to assess their diagnostic sensitivity and specificity. In particular, this study aimed to explore if testing for multiple antibody species can increase the power of RA serodiagnostics by reducing the number of seronegative patients, thereby allowing an earlier diagnosis and treatment of patients negative for the routinely measured antibodies.

## Materials and Methods

### Patients

To determine the sensitivity and specificity of autoantibodies, sera were obtained from 290 RA patients, classified according to the 2010 EULAR/American College of Rheumatology criteria (2). Among these, 165 patients were early RA (inception cohort) receiving their first treatment with methotrexate (MTX) and 125 patients were established RA receiving their first treatment with a TNF inhibitor. The antibody measurements were in general performed at the time of therapy induction or maximum 2 months before or 2 weeks thereafter if a baseline sample was not available (38 patients) because antibody titers are quite stable and seroconversion is rarely observed before or shortly after start of treatment. Disease control samples were collected from 100 patients with osteoarthritis (OA), 50 patients with systemic lupus erythematosus (SLE), 50 patients with ankylosing spondylitis, 13 patients with reactive arthritis (reA), 15 patients with dermatomyositis-polymyositis, 14 patients with granulomatosis with polyangiitis, and 19 osteoporosis patients. In addition, 100 sera from healthy subjects were analyzed for the presence of autoantibodies. Descriptive statistics for the Vienna inception (*n* = 165) and established RA (*n* = 125) cohort are summarized in Table 1. Disease controls had a median age of 55 (43–64) and 68.8% were females. Healthy subjects had a median age of 50 (42.5–55) and 72% were females. An informed consent was obtained from all patients as well as healthy subjects and the study was approved by the ethics committee of the Medical University of Vienna (ethics vote number: 559/2005).

**Table 1:** Descriptive characteristics of rheumatoid arthritis (RA) patients.

	RA (inception)	RA (established)
Age (years)	57.4 (47.2–66.2)	53.2 (44.1–63.3)
Female%	74.2	83.8
Disease duration (years)	**0.1 (0–0.3)**	6.5 (2.5–12)***
Simplified disease activity index	15.6 (9.3–24.2)	14.4 (8.7–21)
Clinical disease activity index	14.2 (8.5–22.1)	12 (7–18.2)
Disease activity score	4.4 (3.4–5.2)	4.2 (3.3–4.8)
C-reactive protein [mg/dl]	0.8 (0.3–1.6)	0.4 (0.2–1.3)
Pain	43 (18–56)	36 (18–56.5)
Patient global disease activity	40 (19.5–60.5)	41 (21–60)
Evaluator’s global disease activity	20 (9.5–35)	21.5 (10–35)
Health access and quality index (HAQ)	**0.4 (0–1)**	0.8 (0.25–1.5)*
Swollen joint count 28	3 (2–6)	3 (2–6)
Tender joint count 28	3 (1–7)	3 (0–6)
Corticosteroids (mg)	6.3 (5–10.5)	6.3 (5–9.8)

Descriptive characteristics of inception and established RA were calculated at treatment start (baseline), i.e., MTX treatment in the inception cohort and anti-TNF treatment in patients with established RA. Values are medians and lower and the upper quartiles provided in brackets. Disease duration was calculated from the date of diagnosis. The corticosteroid dose also refers only to the baseline visit and gives no information about earlier prescriptions. Significant differences between the inception and established RA cohort are shown in bold numbers. Corr. *p*-values for disease duration was ***p < 0.0001 and HAQ was *p = 0.012.

### Detection of Autoantibodies

Serum samples were tested for the presence of IgA, IgG, and IgM isotypes of RF, ACPA, and RA33 by EliA™ platform (Phadia AB, Uppsala, Sweden). Of note, the anti-RA33 EliA™ is a prototype assay that is not yet commercially available (14) but is designed according to the same principle as the commercial assays available for the EliA platform (Phadia AB, Uppsala, Sweden). In this assay, recombinant human hnRNP-A2/B1 expressed in a eukaryotic expression system is used as antigen and a monospecific serum used as standard. According to our established standard protocols, a calibration curve for each isotype is determined during each measurement. This is used to calculate the defined units of the antibodies measured with commercially available assays or the concentration of the antibodies measured with prototype assays (research use only, IgM-CCP, IgA-, IgG-, and IgM-RA33) which have no defined units. Cutoffs for RF IgM, IgA, and IgG as well as ACPA IgG and IgA were employed according to the manufacturer’s instructions. The cutoff for IgG-ACPA used in this study was 7 U/ml, which is (according to the manufacturer) the “equivocal cutoff” while in routine diagnostics the “positive cutoff” of 10 U/ml is used. However, since even at the lower cutoff the assay showed a specificity versus disease controls of 99% also patients with equivocal IgG-ACPA levels were included in our analysis. Cutoffs for prototype anti-RA33 (IgA, IgG, and IgM) and the IgM-ACPA EliA™ were calculated by receiver operating characteristic (ROC) curve analysis (15) against disease controls and healthy subjects. Area under the curve (AUC) values for the ROC curves are shown in Table 2, see Figure S1 in Supplementary Material for the ROC diagrams [made with the R package pROC (16)]. In addition, RF and ACPA had been routinely measured by nephelometry using the N Latex RF kit (employing human IgG as antigen) on a BN II system (Siemens Healthcare GmbH, Germany) and the anti-CCP EliA™ (Thermo Fisher Scientific), respectively.

**Table 2:** Specificity and sensitivity of rheumatoid factor (RF), anti-citrullinated protein antibodies (ACPA), and RA33 isotypes for the diagnosis of rheumatoid arthritis (RA).

	IgA-RF	IgG-RF	IgM-RF	IgA-ACPA	IgG-ACPA	IgM-ACPA	IgA-RA33	IgG-RA33	IgM-RA33	RA33 (total)	IgA-RF/ACPA (total)	RF (routine)	ACPA (routine)
Cutoff	14 IU/ml	28 IU/ml	3.5 IU/ml	7 U/ml	7 U/ml	116.7 µg/l	4.5 µg/l	12 µg/l	32 µg/l			15.9 IU/ml	10 U/ml
Specificity (healthy)	98%	98%	92%	99%	99%	95%	98%	98%	98%	94%	97%	n.d.	n.d.
Specificity (disease controls)	95.3%	98.6%	90%	98.6%	99.4%	95.6%	97.5%	97.2%	95.8%	90%	94.2%	n.d.	n.d.
Patients with RA (*n*)	290	290	290	290	290	290	235	290	243	290	290	290	290
Sensitivity (% positive patients)	50.7%	14.4%	64.8%	34.1%	57.9%	28.6%	6%	6.2%	17.7%	22%	55.2%	59%	55.2%
PPV (healthy)	95.4%	85.4%	86.8%	96.5%	97.9%	82.3%	63.1%	71.6%	84.2%	74.9%	93.7%		
PPV (disease controls)	89.8%	89.3%	84%	95.2%	98.7%	84.1%	57.8%	64.3%	71.7%	64.1%	88.5%		
AUC (RA vs healthy)	0.775	0.643	0.785	0.742	0.754	0.67	0.55	0.608	0.481				
AUC (RA vs disease controls)	0.725	0.643	0.784	0.704	0.777	0.687	0.646	0.47	0.537				

*Antibodies were measured in sera of 290 RA patients, 261 disease controls and 100 healthy subjects. Cutoffs for RF isotypes and for IgA- and IgG-ACPA were used according to the manufacturer’s instructions (U = unit; IU = international unit). Cutoffs for IgM-ACPA and prototype RA33 EliA™ were calculated by receiver operating characteristic (ROC) curve analysis. The total number of RA33 positive patients and IgA-RF and/or IgA-ACPA positive patients are summarized in the last two columns. The data obtained with routinely measured RF (nephelometry) and IgG-ACPA (EliA™) are shown for comparison. Specificity, sensitivity, and positive predictive values are showing the performance of each Ig. The area under the ROC curve (area under the curve [AUC]) values measure the performance without a particular cutoff. An AUC of 0.5 would be random when not choosing the cutoff carefully. The higher an AUC value is, the better is the performance. See also Figure S1 in Supplementary Material for a visualization of the ROCs*.

### Statistical Analysis

For comparison of two groups of numeric values a two-tailed Mann–Whitney *U*-test was performed using R (version 3.2.3). For comparison of two groups of nominal values (gender) Fisher’s exact test was used. If a correction for multiple testing was done (written as “corr. *p*-values”), it was the Bonferroni correction. A *p*-value of <0.05 was considered significant. To distinguish between the *p*-value levels they are depicted as *p ≤ 0.05, **p ≤ 0.01, and ***p ≤ 0.001.

## Results

### Cutoffs, Sensitivities, and Specificities of Autoantibodies

To determine their diagnostic sensitivity and specificity, the nine autoantibody species were measured in 290 patients with RA, 261 disease controls, and 100 healthy subjects. Cutoffs were used either according to the manufacturer or determined by ROC curve analysis (RA33 antibodies and IgM-ACPA). Positive predictive values of antibodies against disease controls or healthy subjects were also calculated. The data are summarized in Table 2. Among the three RF isotypes, IgM-RF showed the highest sensitivity (64.8%) followed by IgA-RF (50.7%) and IgG-RF (14.4%).

Specificities versus disease controls were 95.3% for IgA-RF, 98.6% for IgG-RF, and 90% for IgM-RF, the latter being comparable to the specificity of nephelometric RF (89%) determined in previous studies (11, 17). Of note, testing for IgM-RF proved more sensitive than nephelometric RF determination (64.8 vs 59%; Table 2). Of the 171 patients positive for RF by nephelometry only three were negative by EliA™ and overall the titers of both assays correlated with a coefficient of determination (*R*^2^) of 0.78 and an intraclass correlation of 0.79 (Figure S2 in Supplementary Material). Despite the good correlation, a few discrepant results were obtained which can be explained by methodological differences between the two assays employing antigens (IgG) of human (nephelometry) or rabbit (EliA™) origin which may occasionally cause discrepancies (18).

Among the ACPA isotypes, IgG-ACPA was by far the most sensitive marker (57.9%) and also the most specific one (99.4%). A similar specificity was found for IgA-ACPA (98.6%) which, however, was much less prevalent showing a sensitivity of only 34.1%. IgM-ACPA was the least sensitive (28.6%) and the least specific subtype (95.6%).

To define positivity of RA33 antibodies, cutoffs for prototype RA33 EliA™ were calculated by ROC curve analysis and set to reach at least 95% specificity against disease controls and 98% against healthy subjects. Using these criteria, 4.5 (IgA-RA33), 12 (IgG-RA33), and 32 µg/l (IgM-RA33) were defined as cutoffs for the three anti-RA33 subtypes which showed sensitivities of 6, 6.2, and 17.7%, respectively. Both IgA- and IgG-RA33 showed high specificity (97.5 and 97.2%) while the specificity of IgM-RA33 was 95.8% and thus comparable to IgM-ACPA but superior to IgM-RF (Table 2).

**Figure 1:**
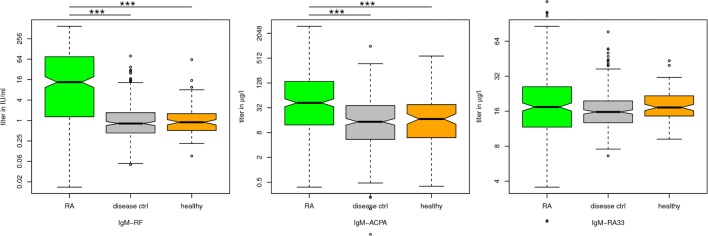
Immunoglobulin (Ig) titers of rheumatoid arthritis (RA) patients vs healthy and disease control patients. The significance corresponds fairly to the overlap of the notches in the boxplots: IgM-rheumatoid factor (RF) and IgM-ACPA are significantly (*p* ≤ 0.001) different between RA patients and disease controls or healthy subjects, whereas IgM-RA33 titers did not significantly differ between the three groups.

Thus, not surprisingly the IgM isotypes of all three antibodies proved less specific than IgG and IgA isotypes. However, of the three measured IgM isotypes (RF, ACPA, and RA33), the titers of IgM-RF and IgM-ACPA were significantly higher in RA patients than in disease controls and healthy subjects (Figure 1). Among the 261 disease controls, false positive results were obtained particularly in patients with OA, ankylosing spondylitis and SLE (Table 3). Since SLE patients are rarely seen in an early arthritis clinic, the number of positive disease controls excluding SLE is additionally indicated in Table 3. Of note, in disease controls, there was hardly any overlap of antibodies with the majority of patients being positive for only one or two antibody species (see added diagnostic value).

**Table 3:** Antibody profile of healthy subjects and disease controls.

	Tested patients	IgA-RF	IgG-RF	IgM-RF	RF total	IgA-ACPA	IgG-ACPA	IgM-ACPA	ACPA total	IgA-RA33	IgG-RA33	IgM-RA33	RA33 total
Healthy	100	2	2	8	11	1	1	5	6	2	2	2	6
SLE	50	11	3	13	18					3	3	6	11
Ankylosing spondylitis	50		1	3	4	3	1	5	8	2	3	5	10
Osteoarthritis	100	3		13	16	1		9	10	1	1	3	6
Reactive arthritis	13	1		1	1	1	1	1	1	1			1
Osteoporosis	19	2	1	3	4			1	1		1	1	2
Dermatomyositis-polymyositis	15			2	2					1	1		2
Granulomatosis with polyangiitis	14			1	1					1	1		2
Total	361	19	7	44	57	6	3	21	26	11	12	17	40
Total excluding SLE	311	8	4	31	39	6	3	21	26	8	9	11	29

*100 healthy subjects as well as 50 patients with systemic lupus erythematosus (SLE), 50 patients with ankylosing spondylitis, 100 patients with osteoarthritis (OA), 13 patients with reactive arthritis (reA), 19 osteoporosis patients, 15 dermatomyositis–polymyositis patients, and 14 patients with granulomatosis with polyangiitis (Wegener’s granulomatosis) were tested for the presence of IgA, IgG, and IgM of RF, ACPA, and RA33. Total numbers of positive patients including or excluding patients with SLE are indicated*.

### Distribution of Autoantibody Isotypes in RA Patients

**Figure 2:**
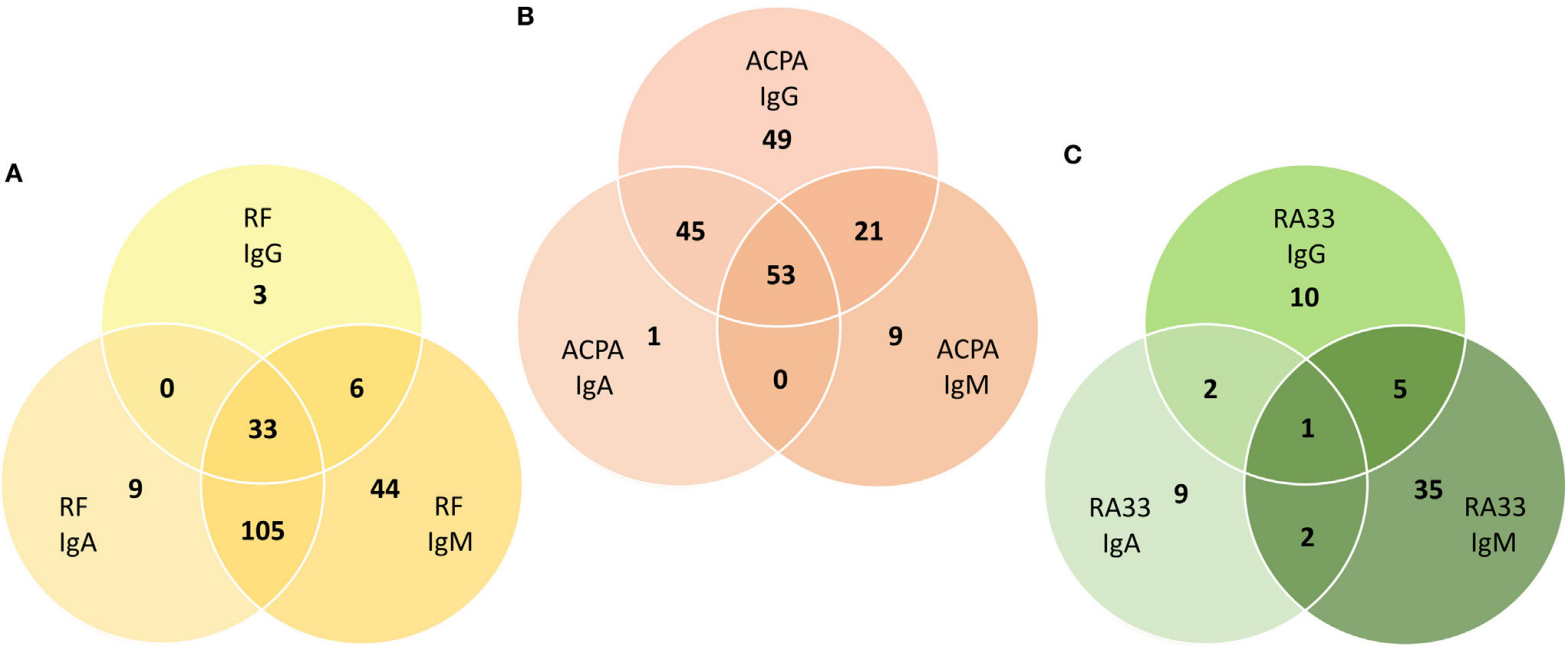
Isotype distribution of rheumatoid factor (RF), anti-citrullinated protein antibodies (ACPA), and RA33 antibodies among antibody positive patients. Venn diagrams visualizing the overlap between **(A)** RF, **(B)** ACPA, and **(C)** RA33 IgA, IgG, and IgM isotypes. The major RF subtype is IgM largely overlapping with IgA and IgG subtypes. The major ACPA subtype is IgG largely overlapping with IgA and IgM subtypes. The major RA33 subtype is IgM but there is little overlap with the IgA and IgG subtypes.

The cohort of 290 RA patients consisted of 165 patients with early RA who had started their first MTX treatment at the Division of Rheumatology and 125 RA patients with established disease who had started their first treatment with TNF inhibitors. No major differences in autoantibody titers were observed between patients with early and established RA (Figure S3 in Supplementary Material). As expected, IgM-RF was the major subtype and also the most sensitive of all nine autoantibody species tested. Thus, 188 patients were positive for IgM-RF, 147 for IgA-RF, and only 42 for IgG-RF. These two isotypes largely overlapped with IgM-RF and only 12 patients were solely positive for either IgA-RF (*n* = 9) or IgG-RF (*n* = 3), while in 44 patients IgM-RF was the only isotype. In total, 200 patients (69%) were RF positive and in 33 patients all three isotypes were detected (Figure 2A).

With respect to ACPA, 168 patients were positive for IgG-ACPA and 99 for IgA-ACPA while IgM-ACPA was found in only 83 patients. Both IgA- and IgM-ACPA largely overlapped with the IgG isotype and only 9 patients were solely positive for IgM- and a single patient was solely positive for IgA-ACPA as compared to IgG-ACPA which in 49 patients was the only ACPA species. In total, 178 patients were ACPA positive and in 53 patients all three isotypes were detected (Figure 2B).

Concerning RA33 antibodies, 14 patients were positive for IgA- and 18 for IgG-RA33. Interestingly, the major RA33 subtype was IgM which was detected in 43 patients. Prevalence of IgG-RA33 was lower but specificity was markedly higher than reported in previous studies (10–13). In total, 64 patients were RA33 positive. However, in contrast to RF and ACPA, the overlap between the RA33 isotypes was marginal (Figure 2C). Thus, only nine patients tested positive for two isotypes and in a single patient co-occurrence of all three isotypes was seen.

### Added Diagnostic Value of Testing for Multiple Isotypes

**Figure 3:**
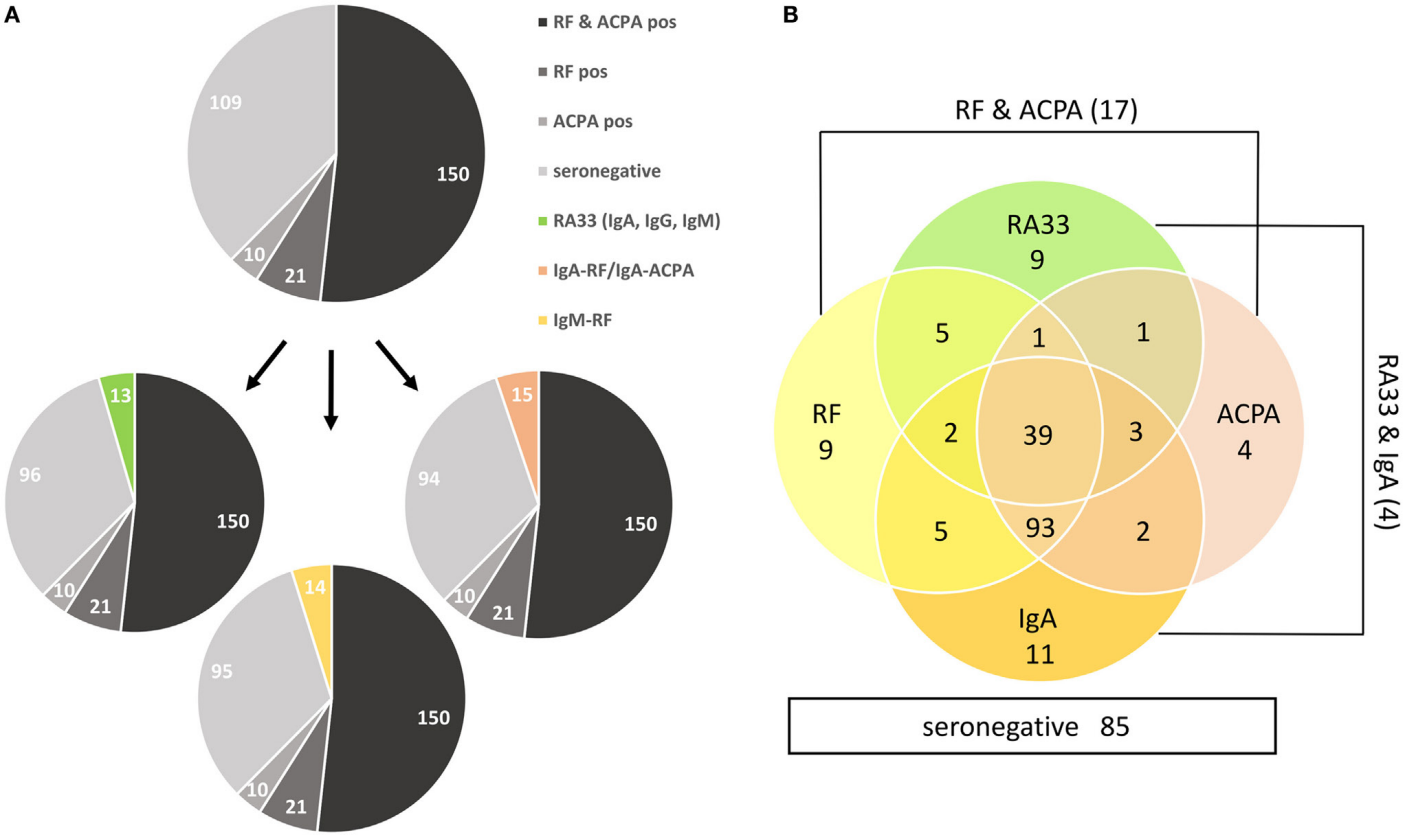
Added diagnostic value of IgA-rheumatoid factor (RF)/anti-citrullinated protein antibodies (ACPA), IgM-RF and RA33 antibodies. **(A)** Numbers of patients tested positive by routine diagnostics (RF nephelometry, IgG-ACPA) and seronegative patients (upper pie chart) showing additional RA33 antibodies (lower left pie chart), IgM-RF (lower middle pie chart) or IgA-RF/ACPA (lower right pie chart). **(B)** Venn chart visualizing the diagnostic overlap of RA33 and IgA antibodies with routine diagnostics (RF nephelometry, IgG-ACPA). The numbers of seronegative patients (*n* = 85), RF, and IgG-ACPA positive but RA33 and IgA-RF/ACPA negative patients (*n* = 17) as well as patients positive for both RA33 and IgA-RF/ACPA but negative for RF and ACPA (*n* = 4) are also indicated.

Among the 290 RA patients, 150 (51.7%) were positive for both RF and ACPA by routine diagnostics, while 21 (7.2%) and 10 (3.4%), respectively, were solely positive for either RF or ACPA (Figure 2A); 109 (37.6%) patients were negative for both antibodies and are therefore referred to as seronegative. Out of these, 13 (4.5%) patients showed at least one RA33 subtype and 15 (5.2%) patients were positive for either IgA-RF (*n* = 14) and/or IgA-ACPA (*n* = 4), whereas IgG-RF and IgM-ACPA did not further increase the sensitivity of autoantibody diagnostics. In addition, 14 (4.8%) RF negative patients (as determined by nephelometry) tested positive for IgM-RF by EliA™ (Figure 3A).

The majority of antibody positive RA patients was found to be triple positive for (nephelometric) RF, IgG-ACPA and either IgA-RF or IgA-ACPA. Interestingly, among the 64 RA33 positive patients 48 were also positive for IgA-RF and/or IgA-ACPA (Figure 3B). Concerning the added diagnostic value of testing for multiple antibodies, 24 (8.3%) formerly seronegative patients were positive for either IgA-RF/ACPA or RA33 antibodies. Therefore, additional testing for IgA-RF/ACPA and RA33 reduced the number of seronegative patients by approximately 22% (Figure 3B). Additional specification of IgM-RF further reduced the number of seronegative patients resulting in a total reduction of 30%. Importantly, with respect to the diagnostic value of the IgM-RF determination, the majority of patients negative for nephelometric RF but positive for IgM-RF were low-titered but showed additional reactivities (including IgG-ACPA), in contrast to the controls which were usually monospecific for IgM-RF.

**Figure 4:**
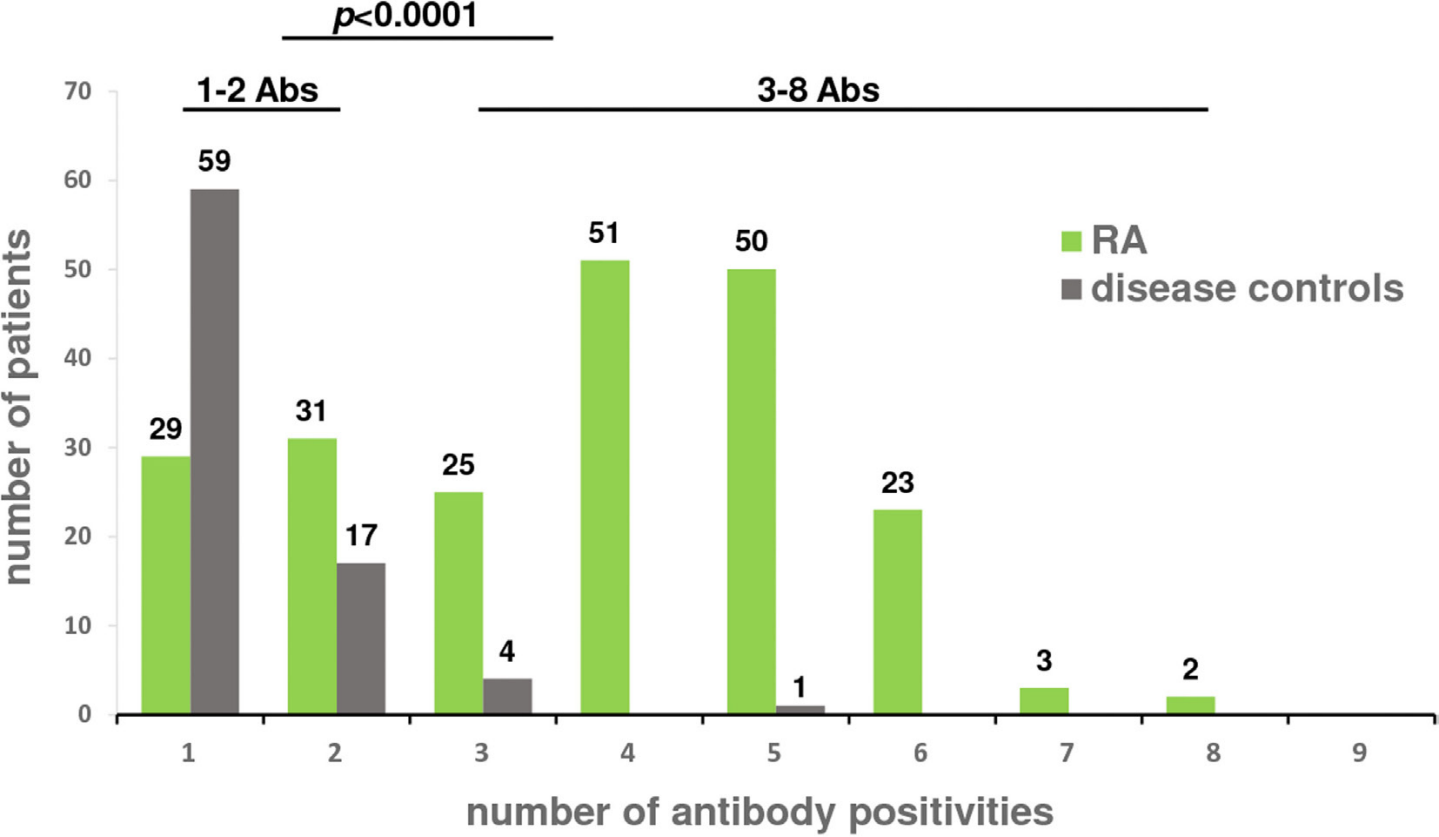
Number of antibody positivities in rheumatoid arthritis (RA) patients and disease controls. 290 RA patients and 261 disease controls were tested for the presence of IgA, IgG, and IgM isotypes of rheumatoid factor, anti-citrullinated protein antibodies and RA33. The number of antibodies detected in a patient’s serum is indicated on the *x*-axis; the number of patients is indicated on the *y*-axis. Disease controls were found to have significantly (*p* < 0.001) fewer antibody positivities (1–2 Abs) compared to RA patients who commonly had more than two antibodies (3–8 Abs).

Typically, RA patients were positive for multiple antibody species, which was in sharp contrast to the disease controls (Table 3). Among the 81 antibody positive disease controls, 73% showed only one antibody species whereas the majority of antibody positive RA patients (74%) had at least three antibodies with only 14% of the patients showing singular positivities, mostly of the IgM or IgA isotype (Figure 4). Thus, the presence of three antibodies had a specificity of 94% and the presence of four antibodies was almost 99% specific for RA, even in IgG-ACPA negative patients among which eight showed four or more antibodies and four were triple positive. Double positive disease control usually showed only IgM antibodies with the notable exception of SLE patients in which IgM-RF and IgA-RF commonly occurred together, whereas none of them showed any ACPA isotype.

There were no major differences in clinical parameters between antibody negative and positive patients, except that TJC28 is significantly (*p* = 0.04) lower in patients with 4 or more antibodies compared to seronegative patients (Table 4).

**Table 4:** Clinical parameters of rheumatoid arthritis patients with none, 1–3, and 4 or more positive Igs.

	Seronegative	1–3 antibodies	4 or more antibodies
Number of patients	78	88	124
Age (years)	53.4 (44.2–65.7)	57.2 (46.8–66.3)	56.6 (46.6–63.6)
Female%	80.8%	85%	75%
Disease duration (years)	0.3 (0.025–2.1)	0.5 (0–6.1)	1 (0.1–8.3)
Simplified disease activity index	16.6 (9.9–25.1)	13.6 (9.3–23.3)	14.8 (8.7–22)
Clinical disease activity index	14.9 (9.2–22.7)	12 (8.5–20.2)	12.7 (7.4–19.25)
Disease activity score	4.4 (3.7–5.1)	4.3 (3.1–5.1)	4.2 (3.4–5)
C-reactive protein [mg/dl]	0.5 (0.2–1.2)	0.5 (0.2–1.3)	0.8 (0.3–1.7)
Pain	41 (22–58)	40 (20.5–60.5)	35 (15–52)
Patient global disease activity	40 (23–58)	45 (20–61.5)	38 (19–60)
Evaluator’s global disease activity (EGA)	21 (10–34)	20 (10–29.5)	24 (10–38)
Health access and quality index	0.8 (0.125–1.5)	0.8 (0.25–1.25)	0.6 (0.125–1.1)
Swollen joint count 28	3 (2–4)	3 (2–5)	4 (2–7)
Tender joint count 28	**4 (2–8)**	3 (0–7)	2 (0–5)*
Corticosteroids (mg)	6.3 (5–12.5)	6.3 (5–10)	6.3 (5–6.3)

Values are medians and lower and the upper quartiles are indicated in brackets. Disease duration was calculated from the date of diagnosis. TJC28 was found to be significantly (*corr. *p* = 0.04) lower in patients with 4 or more antibodies compared to seronegative patients.

## Discussion

Approximately one-third of RA patients are commonly negative for RF and ACPA, the two serological marker antibodies which are routinely determined in RA serodiagnostics. However, it is still not fully clarified whether these patients are completely negative for autoantibodies or may rather generate antibody species that are not covered by routine diagnostics where usually IgG-ACPA (by ELISA) and RF (by nephelometry or ELISA) are determined. This issue has been in addressed in several previous studies in which especially IgA subtypes of RF and ACPA were found to occur mainly in seropositive patients. However, in none of these studies, all three ACPA and RF isotypes were investigated in parallel and disease controls were usually not included (4–6, 8). The results obtained in our comprehensive analysis in which three isotypes of RF, ACPA, and RA33 were determined in a large number of RA patients, disease controls, and healthy subjects (651 individuals in total) show that about one-third of “seronegative” patients generate antibodies known to be highly associated with RA including IgA isotypes of RF and ACPA as well as RA33 antibodies. Although RA33 antibodies were much less prevalent than the other antibodies and also proved less specific, they nevertheless contributed to the reduction of the serological gap being present in 12% of seronegative patients usually in conjunction with other antibody species. A similar number of seronegative patients were positive in the IgM-RF assay which proved slightly more sensitive than the nephelometric assay (Figure S2 in Supplementary Material). Most of the additionally detected IgM-RF were of low titer but usually co-occurred with other antibody species, in contrast to (seropositive) healthy or disease controls in which IgM-RF was usually the only species. Only three sera positive by nephelometry were negative in the IgM-RF assay (Figure S2 in Supplementary Material), which may be explained by the use of antigens from different species (18). Of note, most of the seronegative RA patients showed multiple reactivities, whereas controls were usually monospecific for a single antibody species and showed predominantly one or two IgM reactivities, particularly patients with OA or ankylosing spondylitis. While low titer IgM antibodies occurring as single entities are of limited diagnostic usefulness they can nevertheless be helpful markers when co-occurring with other antibody species such as IgA-RF/ACPA isotypes or RA33 antibodies, which proved also less specific for RA than IgG-ACPA.

IgG-RA33 has been described in several studies to have a prevalence of 20–30% and a specificity of approximately 90% (13, 19). Although in our cohort the newly developed IgG-RA33 EliA™ prototype proved less sensitive, specificity was better than 97% and similar values were obtained for IgA-RA33. However, due to the modest sensitivity, the positive predictive values of RA33 antibodies were lower than those of the other antibodies investigated (Table 2). Interestingly, IgM-RA33 was the most prevalent RA33 subtype and showed similar specificity as IgM-ACPA while IgM-RF was the least specific of all nine isotypes investigated. In contrast to RF and ACPA, RA33 isotypes showed little overlap and therefore, despite their modest sensitivities, RA33 antibodies were seen in more than 20% of RA patients including, as mentioned above, a substantial number of seronegative ones. Importantly, RA33 antibodies commonly co-occurred with other antibody species such as IgM-RF or IgM-ACPA, which are also less specific than IgG-ACPA.

Remarkably, co-occurrence of these antibodies was very specific for RA and not observed in disease controls or healthy subjects. In fact, co-occurrence of IgM-RA33 with other antibodies was seen in only 8 out of 261 disease controls and in a single healthy subject. Hence, determination of RA33 antibodies and RF/ACPA isotypes, especially IgA- and IgM-RF, reduced the number of seronegative patients by approximately 30%. Another important aspect of this study is the observation that sera of RA patients generally contained multiple reactivities, in contrast to sera from disease controls and healthy subjects. However, no major differences in clinical parameters were seen at baseline between seronegative patients, patients with 1–3 antibodies and patients with 4 or more antibodies—a pattern highly specific for RA, even in the absence of IgG-ACPA—except that TJC28 was significantly lower in patients with 4 or more antibodies compared to seronegative patients. We have not yet analyzed disease progression and outcome or response to therapy but it has recently been shown by other investigators that patients with multiple antibodies are at increased risk for relapse when tapering DMARD therapy (20, 21).

Therefore, the determination of multiple antibodies and isotypes, even if they are not highly specific for RA, does not only reduce the number of seronegative (i.e., IgG-ACPA and RF negative) patients but seems to have also prognostic value with respect to disease progression and response to therapy, a matter that is currently under investigation. Thus, it is conceivable that patients with a high number of autoantibodies and hence a high level of autoimmunity may be more responsive to therapies targeting B- and T-lymphocytes such as the anti-CD20 antibody rituximab or the CTLA4-Ig construct abatacept (22). In this sense, it is certainly worthwhile to search for additional antibodies (23) or subtypes (24) and other disease markers (including miRNAs and genetics) that together may help to further stratify RA patients with the aim to treat them more efficiently in a personalized way.

## Conclusion

Testing for multiple autoantibody specificities adds diagnostic value in covering more RA patients and reducing the diagnostic gap left by routine RF and IgG-ACPA determination. Furthermore, the number of antibodies co-occurring in a patient’s serum might provide some prognostic value allowing further subclassification of patients.

## Ethics Statement

This study was carried out in accordance with the recommendations of the “guidelines for storing and using patients samples” of the Ethical Committee of the Medical University of Vienna. With written informed consent from all subjects. All subjects gave written informed consent in accordance with the Declaration of Helsinki that their blood and urine samples can be stored and used for scientific purposes. The protocol was approved by the Ethical Committee of the Medical University of Vienna (ethics vote number: 559/2005).

## Author Contributions

All authors participated in drafting the article or revising it critically for important intellectual content, and all authors gave final approval of the version to be submitted. Study conception and design: DS, AP, and GS. Acquisition of data: DS, AP, HH, MG, SS, and TH. Analysis and interpretation of data: DS, AP, FA, PS, SB, JS, and GS.

## Conflict of Interest Statement

MG, SS, and TH are employees of Thermo Fisher Scientific—Phadia GmbH. All other authors declare that the research was conducted in the absence of any commercial or financial relationships that could be construed as a potential conflict of interest.
