# Supplementary material for: Determination of Autoantibody Isotypes Increases the Sensitivity of Serodiagnostics in Rheumatoid Arthritis
Source: Front Immunol. 2018 Apr 24;9:876. doi: 10.3389/fimmu.2018.00876 (PMC5929149; doi:10.3389/fimmu.2018.00876)
Supplement: Supplementary file 1 [file Presentation_1.PDF]

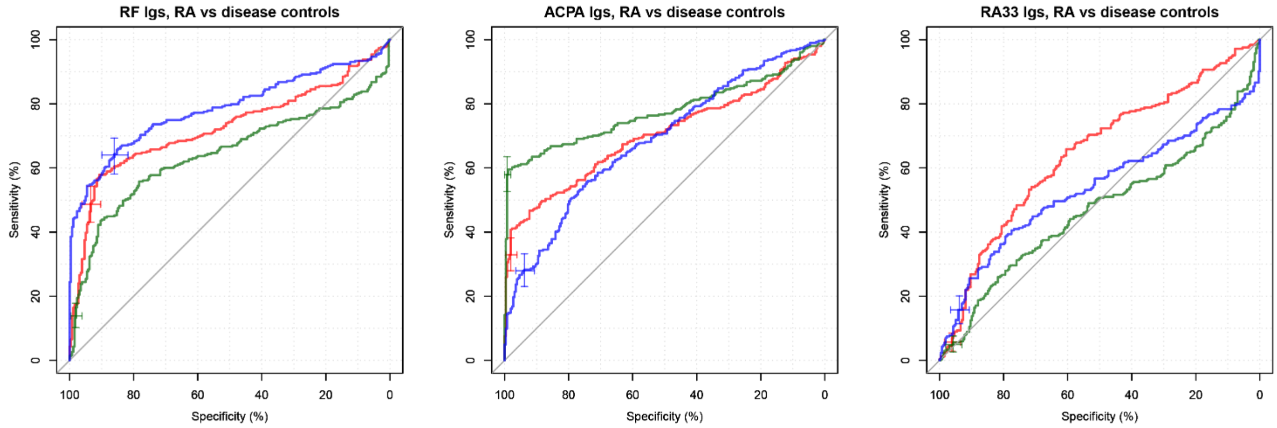

Suppl. figure 1 ROC curves to assess discriminatory power between rheumatoid arthritis (RA) and disease controls, with titers of IgA (red), IgG (green) and IgM (blue) of rheumatoid factor (RF), anti-citrullinated protein antibodies (ACPA) and RA33. The horizontal and vertical bars (the crosses) are the 95% confidence intervals for the selected cutoffs (see also table 2 for numbers).
