# Supplementary material for: Determination of Autoantibody Isotypes Increases the Sensitivity of Serodiagnostics in Rheumatoid Arthritis
Source: Front Immunol. 2018 Apr 24;9:876. doi: 10.3389/fimmu.2018.00876 (PMC5929149; doi:10.3389/fimmu.2018.00876)
Supplement: Supplementary file 2 [file Presentation_2.PDF]

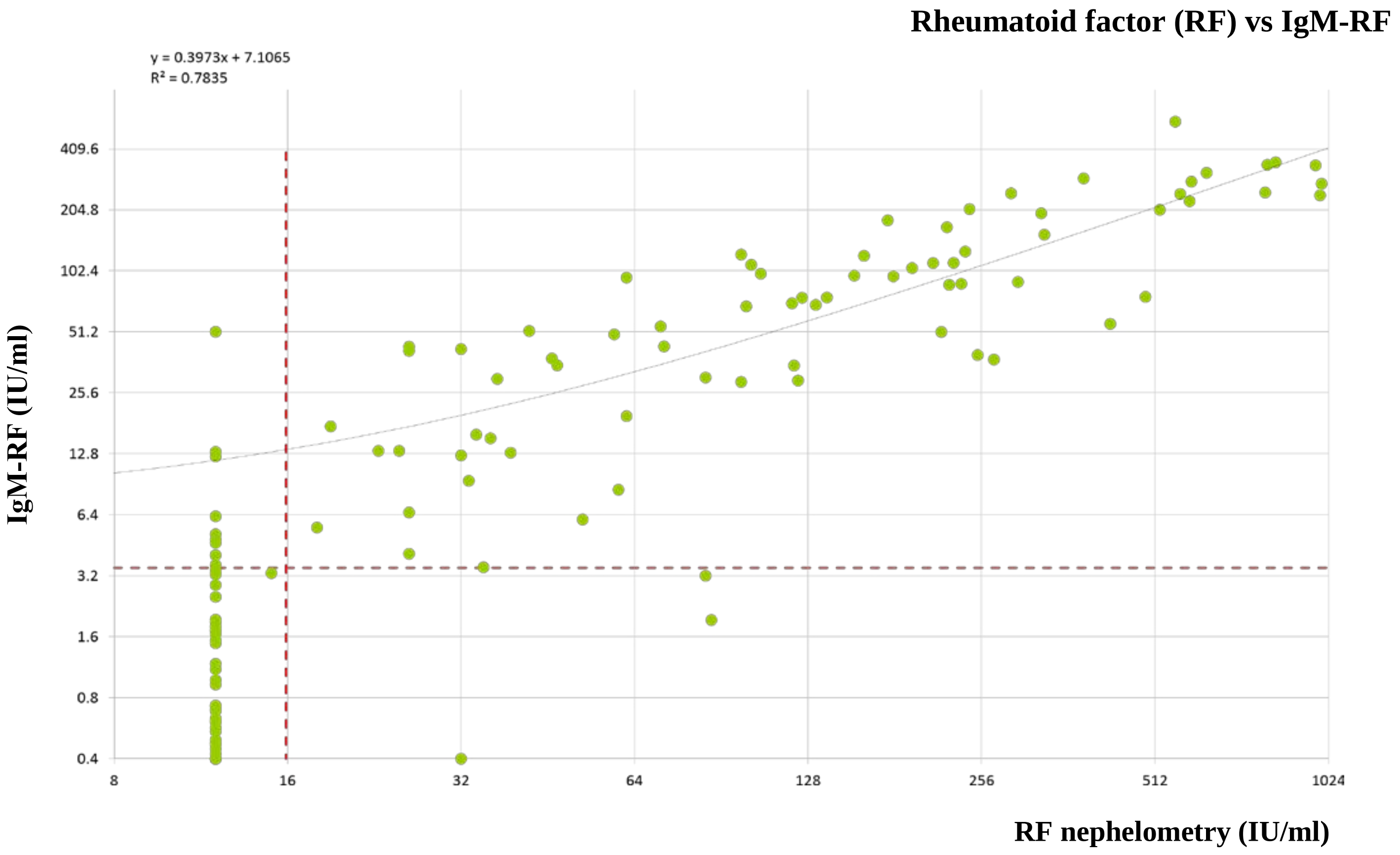

Suppl. figure 2 Correlation between titers of IgM-rheumatoid factor (RF) and RF measured by nephelometry. Both axis ticks show the untransformed values, the drawing scale is log transformed. The minimal measured value for RF by nephelometry is 12 IU/ml, the minimal measured value for IgM-RF is 0.4 IU/ml. The diagnostic cutoff values for the assays are 16 IU/ml for nephelometry and 3.5 IU/ml for IgM-RF (indicated by dotted lines). The equation and the coefficient of determination/R2 are stated in the upper left corner.
