# Supplementary material for: Determination of Autoantibody Isotypes Increases the Sensitivity of Serodiagnostics in Rheumatoid Arthritis
Source: Front Immunol. 2018 Apr 24;9:876. doi: 10.3389/fimmu.2018.00876 (PMC5929149; doi:10.3389/fimmu.2018.00876)
Supplement: Supplementary file 3 [file Presentation_3.PDF]

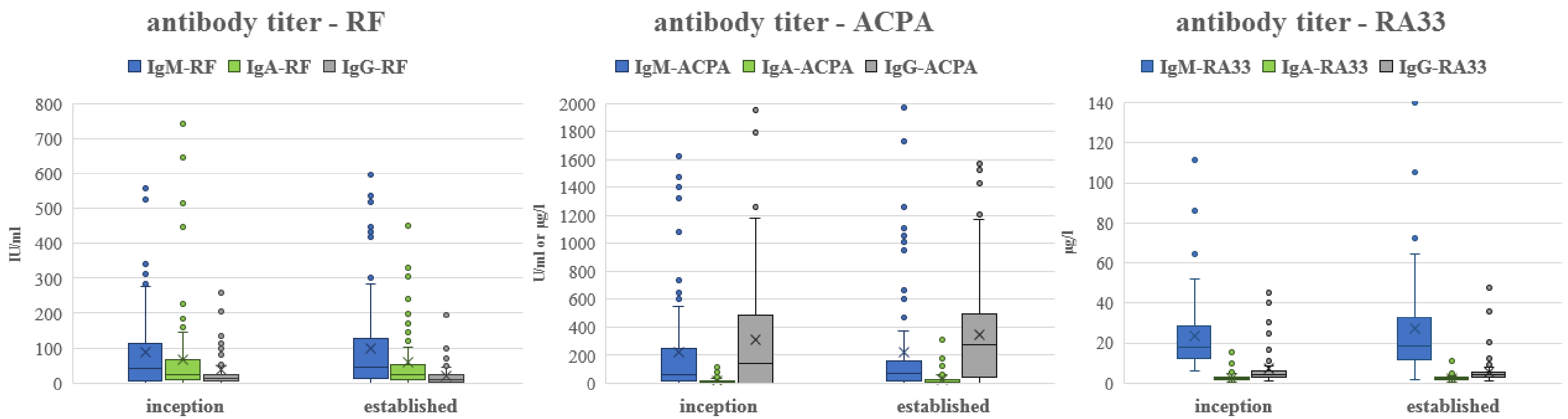

Suppl. figure 3 Antibody titer in inception and established rheumatoid arthritis (RA).
Antibody titers of rheumatoid factor (RF), anti-citrullinated protein antibodies (ACPA) and RA33 of patients starting their first therapy with MTX (inception) was compared to patients with established disease starting their first treatment with a TNF inhibitor. Statistical significance was calculated by two-tailed Mann-Whitney U test with Bonferroni correction. There was no significant difference between the two groups regarding the titers of antibodies. The mean value is indicated by an x.
